# ﻿*Polygalaqii*, a new species of Polygalaceae from limestone landform in Southern Hunan, China

**DOI:** 10.3897/phytokeys.244.121759

**Published:** 2024-07-09

**Authors:** Ang Liu, Jian-jun Zhou, Xiong Li, Xun-lin Yu, Lei Wu

**Affiliations:** 1 Central South University of Forestry & Technology, Changsha 410004, Hunan, China Central South University of Forestry and Technology Changsha China; 2 Hunan Agriculture and Forestry Industry Survey and Design Institute Co., Ltd, Changsha 410007, Hunan, China Hunan Agriculture and Forestry Industry Survey and Design Institute Co., Ltd Changsha China; 3 Guangxi Forestry Inventory and Planning Institute, Nanning 530011, Guangxi, China Guangxi Forestry Inventory and Planning Institute Nanning China

**Keywords:** Hunan province, limestone landform, new species, *Polygala*, taxonomy

## Abstract

*Polygalaqii*, a new species, is described and illustrated from limestone landform in southern Hunan, China. The new species resembles *P.fallax* and *P.arillata* in flower structure of the plants, but readily differs from the latter two in having erect and shorter inflorescences (0.2–1cm VS 10–15cm VS 7–10cm), and fewer flowers (1–5 flowers VS 10–30 flowers VS 10–20 flowers), and the latter two have a later flowering period (late March to mid-April VS May to August VS May to October). And it is an extremely unique new species that will hibernate in the hot summer of July and August. Following the IUCN Red List Criteria, *P.qii* is assessed as ‘Data Deficient (DD)’.

## ﻿Introduction

There are approximately 500 species in the *Polygala* L. (1753:701) (Polygalaceae), which are almost globally distributed. The Flora of China includes 44 species, of which 21 are endemic to China (Chen et al. 1997; Chen et al. 2008; APG 2016). The roots of some plants in the genus *Polygala* can be used as medicinal materials, such as *P.fallax*.

In March 2020, during our investigation in the limestone area of southern Hunan, we discovered a unique species of *Polygala* plant which they grew in the crevices of dry limestone. The plant is different from all the plants of the Polygala genus recorded in Hunan Province; for example, it is a deciduous shrub, blooms very early (usually in late March), leaves have membranous transparent edges, and fewer but denser flowers on the inflorescence and so on. In August of the same year, when we visited the area again to investigate, we found that this plant had already fallen leaves.

In the following year, we collected more specimens of this species, and through phenological observation and morphological research, we finally confirmed that this is a new species.

## ﻿Material and methods

The specimens are mainly stored in the Herbarium of Forest Plants in Central South University of Forestry and Technology (CSFI). Morphological observations of the new species were derived from field observations, as well as study of specimens. The conservation status of this new species is based on field observations in accordance with IUCN Red List guidelines (IUCN 2022).

## ﻿Taxonomic treatment

### ﻿ Polygala qii

Taxon classificationPlantaeFabalesPolygalaceae

X.L.Yu, J.J.Zhou & A.Liu sp. nov.

9EDE3CF2-FA4F-5AD2-A822-7D5B72BE7344

urn:lsid:ipni.org:names: 77344993-1

Figs 1 , 2

#### Diagnosis.

This new species is similar to *P.fallax* Hemsl. and *P.arillata* Buch.-Ham. & D. Don, but it differs from the latter two in having erect and shorter inflorescences (0.2–1cm VS 10–15cm VS 7–10cm) and fewer flowers (1–5 flowers VS 10–30 flowers VS 10–20 flowers). Its caruncle is foam, and the latter two are helmeted in shape. Please refer to Fig. 3 and Table 1.

**Table 1.** Comparison of morphological characters among *Polygalaqii*, *P.fallax* and *P.arillata*.

Characters	*Polygalaqii* sp. nov.	*P.fallax*	*P.arillata*
Plants	0.5–1.5m	1–3m	1–5m
Leaves	5–11 × 2–5cm	8–20 × 4–6.5cm	6.5–14 × 2–2.5cm
Racemes	erect	drooping	drooping
	0.2–1cm, to 5cm at fruiting	10–15cm, to 30cm at fruiting	7–10cm, to 30cm at fruiting
	1–5 flowers	10–30 flowers (or more)	10–20 flowers (or more)
Flowers	yellowish white, apex with purplish red	yellow	yellow, or apex with orange red
Caruncle	foam	helmeted	helmeted
Flowering period	late March to mid-April	May to August	May to October

**Figure 1.**
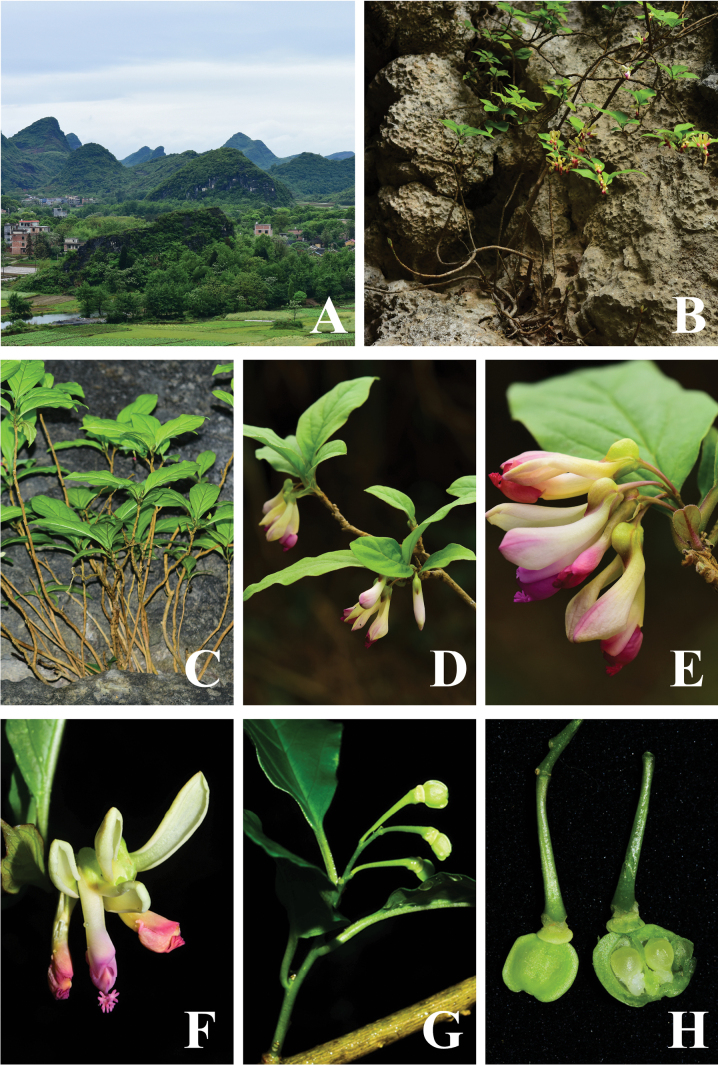
*Polygalaqii* sp. nov. **A** habit **B** plant **C** plant **D** branch with flowers **E** inflorescence **F** front view of flowers **G** infructescence **H** capsules and Seeds. Photographed by Ang Liu.

**Figure 2.**
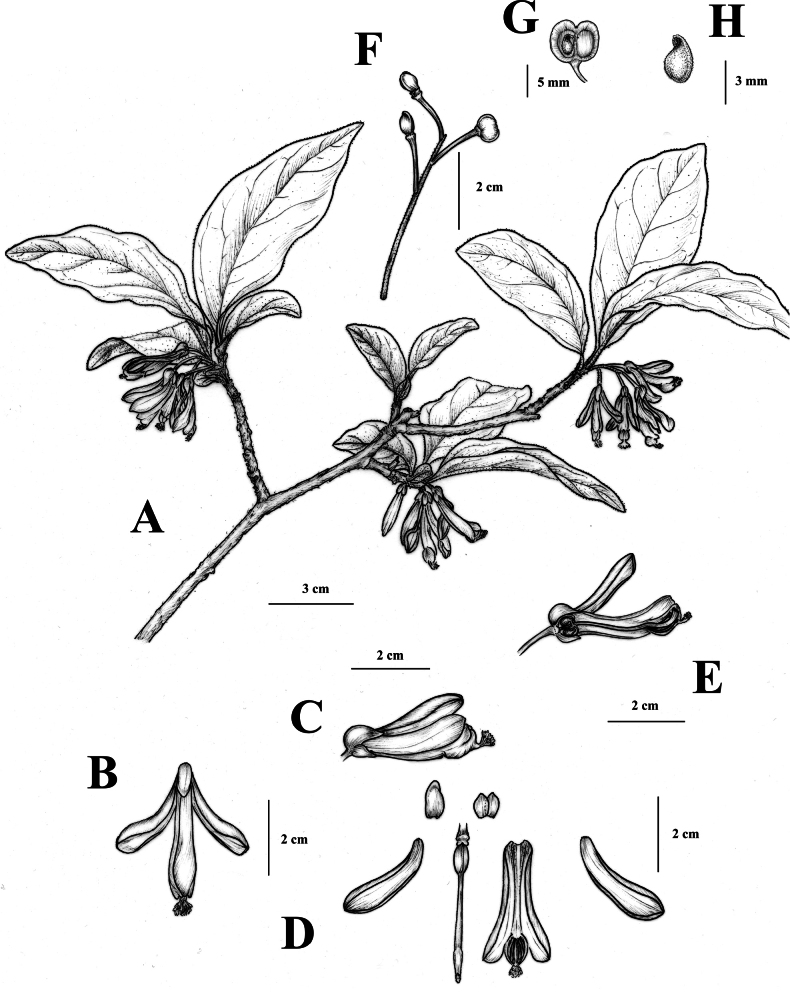
*Polygalaqii* sp. nov. **A** branches with flowers **B** top view of flower **C** side view of flower **D** anatomical structure of flower, sepals, petals and pistil **E** longitudinal section of flower **F** infructescence **G** capsule **H** seed. Drawn by PhD Jing Tian.

**Figure 3.**
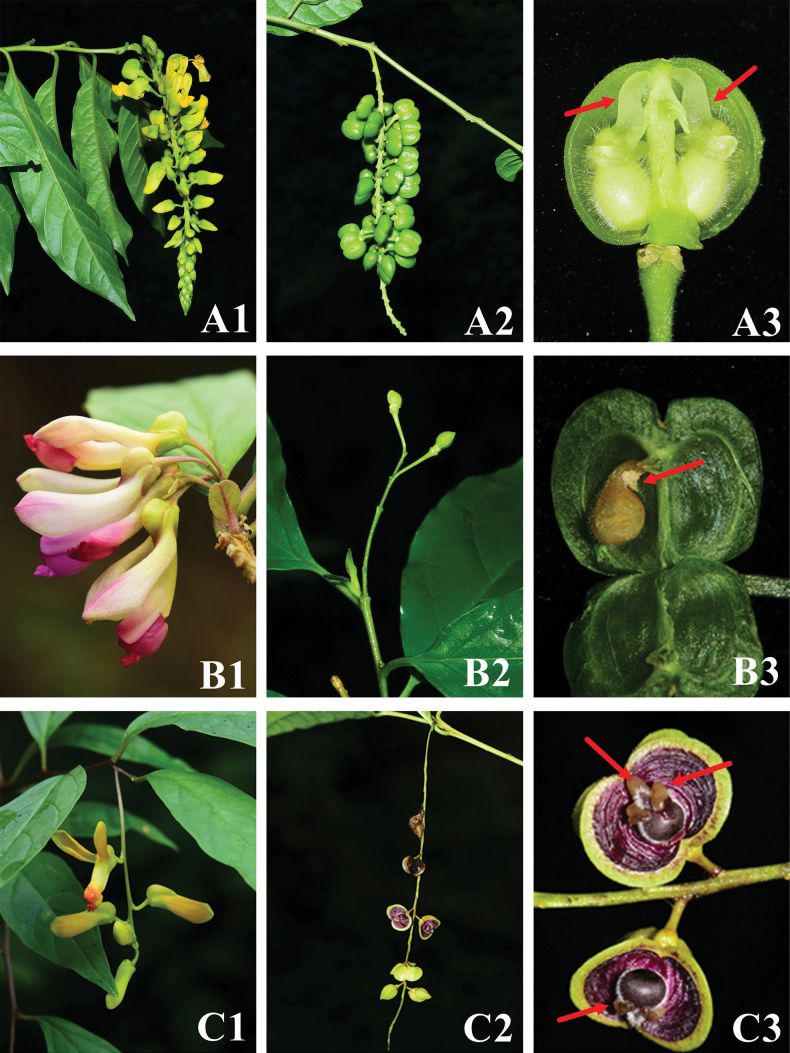
Morphological comparison between *Polygalafallax* (**A1–A3**), *Polygalaqii* sp. nov. (**B1–B3**), *Polygalaarillata* (**C1–C3**). **A1, B1, C1** inflorescence **A2, B2, C2** infructescence **A3, B3, C3** seeds, the red arrow represents caruncle. **C2, C3** photographed by Xin-xin Zhu, others photographed by Ang Liu.

#### Type.

**Figure 4.**
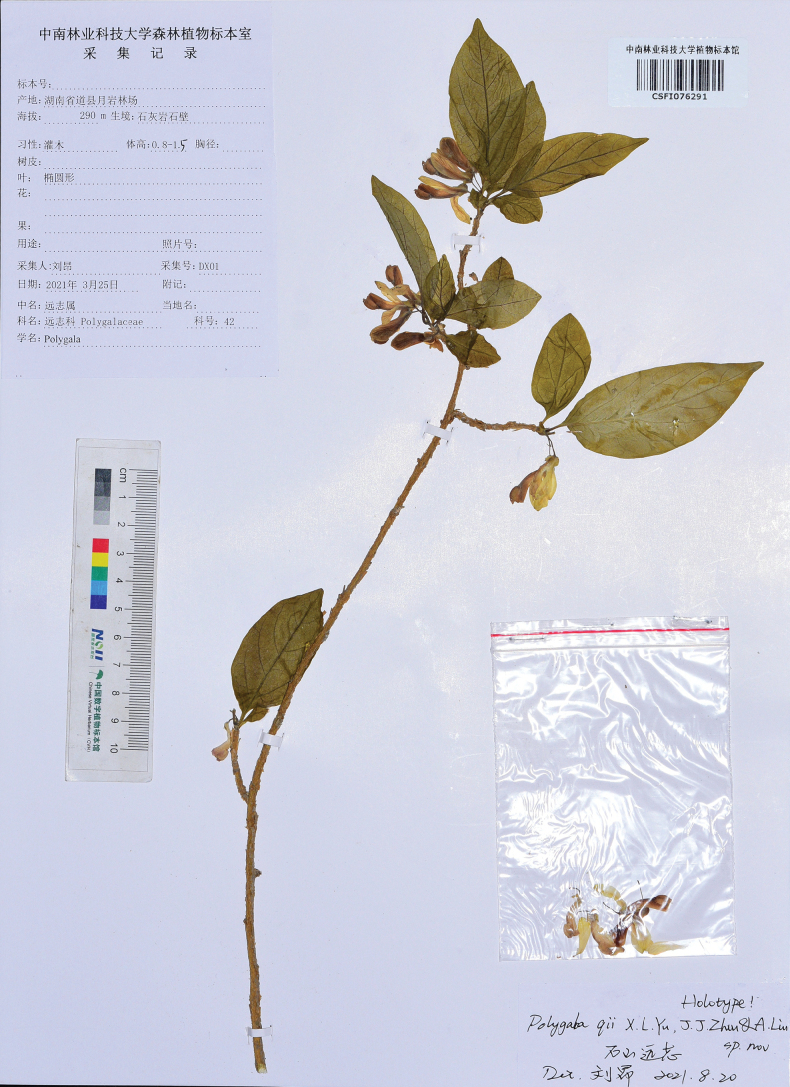
Holotype of *Polygalaqii* sp. nov. (*Ang Liu* DX01, CSFI 076291).

China. Hunan: Yongzhou City, Dao County, Yueyan Forest Farm, in the crevices of dry limestone, elevation ca. 290 m, 25 March 2021, *Ang Liu* DX01 (Holotype CSFI!, isotype CSFI!, HIB!). Please refer to Fig. 4.

#### Description.

Shrubs, 0.5–1.5m high. ***Branchlets*** yellow, sparsely pilose, and the branch bark is cracked into irregular thin scales, especially on the specimens. ***Leaves*** alternate, clustered at the top of branchlets. ***Petiole*** ca. 1 cm, pubescent. ***Leaf blade*** papery, elliptic to oblong elliptic, 5–11 × 2–5cm, with membranous transparent edge, full margin, ciliate, both sides sparsely pubescent, dense along the veins, then gradually glabrous, midvein raised abaxially, depressed adaxially, lateral veins 5 or 6 pairs, apex acuminate or short tail tip, base cuneate or obtuse. ***Racemes*** opposite to leaves, with 1–5 flowers, erect or slightly drooping at the apex, densely pubescent, 0.2–1cm long, up to 5cm at fruiting. ***Pedicel*** glabrous, ca. 0.5cm long, to 1cm at fruiting. ***Flowers*** 1.6–2.3cm. ***Sepals*** 5, ciliate, fall off after flower, outer 3 small, unequal in size, upper 1 deep pocket shaped, 0.5cm long, lateral 2 oval, ca. 0.3cm long, inner sepals 2, petal shaped, yellowish white, obliquely obovate, edge rolled in a boat shape, 1.5–2cm, and at right angles to the petals. ***Petals*** 3, connate in lower 2/3, yellowish white, with light purplish red at the apex, slightly fleshy; keel longer than lateral petals, apex with multifid appendages, appendages with short stalks at the base, ca. 0.15cm. ***Stamens*** 8; filaments ca. 1.5cm, lower 2/3 united, forming an open staminal sheath, adnate with petals; anthers ovoid. Disk fleshy. ***Ovary*** round, flattened, ca. 0.4cm, glabrous, style ca. 1.5cm, bent to the top, with knee bending at 2 / 3, and obviously expanded to the apex in a trumpet shape. ***Capsules*** green, baccate, broadly reniform or slightly cordate, ca. 1 cm, margin winged, ciliate, apex emarginate, mucronate. ***Seeds*** globose, sparsely white pubescent, caruncle foam.

#### Phenology.

Flowering from late March to mid-April; fruiting from late April to early May. What is very special is that this new species enters a dormant period with leaf withering in mid-July.

#### Etymology.

The new species is named after Professor Cheng-jing Qi (CSUFT&CSFI), who has made great contributions to the study of Hunan flora (Qi & Yu, 2002).

#### Vernacular name.

The Chinese name of the new species is ‘石山远志’,and the pronunciation of the Chinese Pinyin is ‘shí shān yuǎn zhì’.

#### Distribution and habitat.

This new species is currently only found in the limestone landform areas of Dao County and Ningyuan County, which usually grows in the crevices of dry limestone.

#### Additional specimens examined

**Figure 5.**
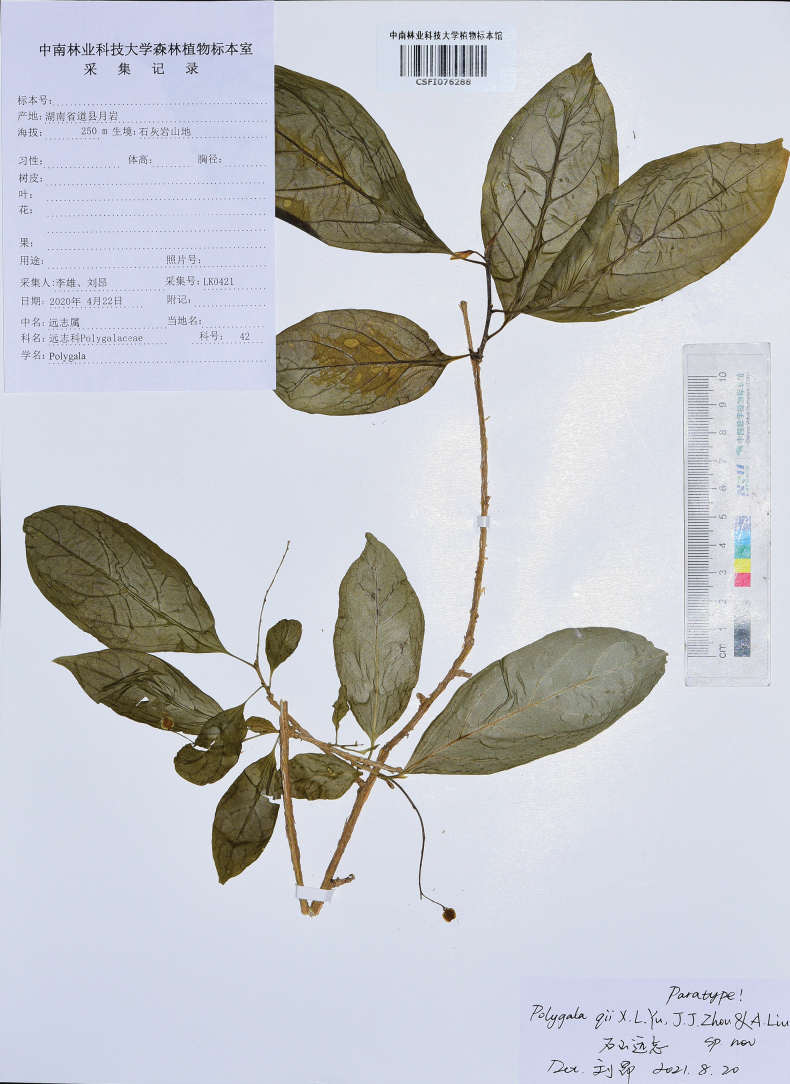
Paratype of *Polygalaqii* sp. nov. (*Xiong Li & Ang Liu* LK0421, CSFI 076288).

**Figure 6.**
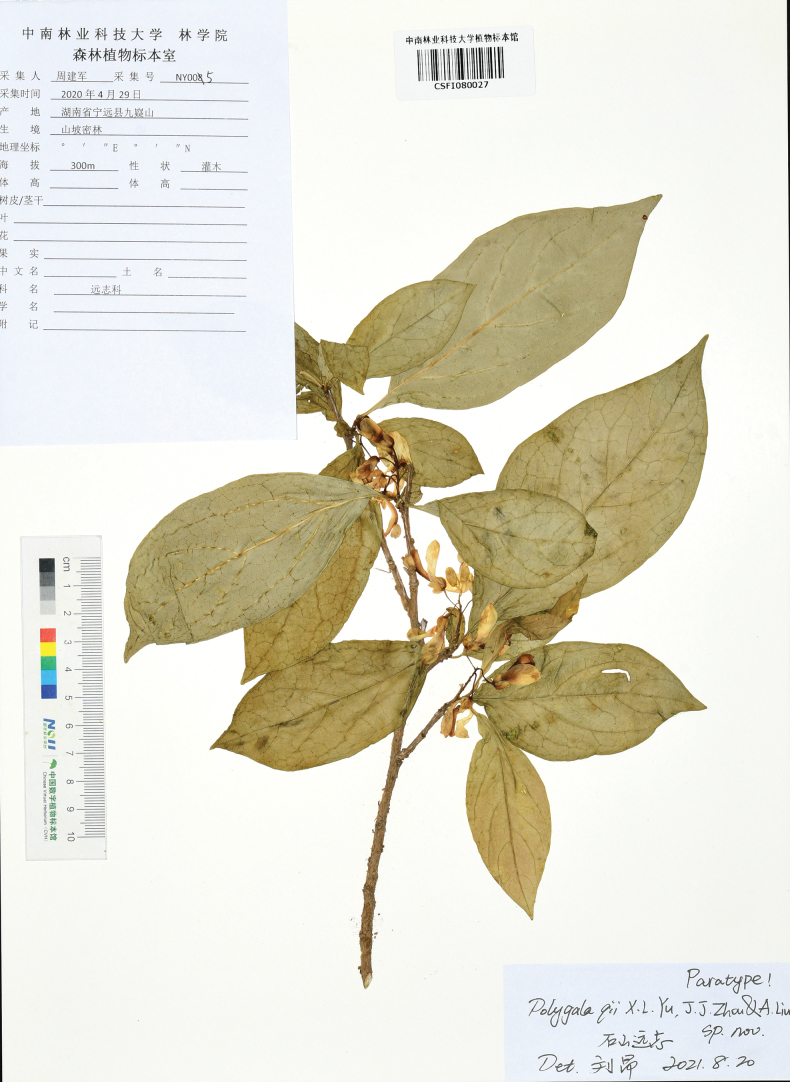
Paratype of *Polygalaqii* sp. nov. (*Jian*-*jun Zhou* NY005, CSFI 080027).

**(Paratypes).** China. Hunan: Yongzhou City, Dao County, Yueyan Forest Farm, in the crevices of dry limestone, elevation ca. 250 m, 22 April 2020, *Xiong Li &Ang Liu* LK0421(CSFI!, HIB!&CSH!); Yongzhou City, Ningyuan County, Jiuyi Mountain, in the crevices of dry limestone, elevation ca. 300 m, 29 April 2020, *Jian*-*jun Zhou* NY005(CSFI!). Please refer to Figs 5, 6.

#### Conservation status.

At present, we have only found two populations with a total of about 30 individuals in the limestone areas of Dao County and Ningyuan County. However, there are vast limestone landforms in southern Hunan, and there may be distribution of this new species in these areas. Of course, we need a broader and deeper investigation to confirm that. According to the IUCN red list criteria (IUCN 2022), the conservation status of the new species should be better categorized as ‘Data Deficient (DD)’.

## ﻿Discussion

We have previously discovered some new species of *Primulina* in the limestone areas of southern Hunan, such as *P.jiangyongensis* X. L. Yu & Ming Li (Li et al. 2014), *P.porphyrea* X. L. Yu & Ming Li (Li and Yu 2015), *P.cataractarum* X. L. Yu & A. Liu (Ding et al. 2021) and *P.gracilipes* X. L. Yu & A. Liu (Gong et al. 2022) and so on. The discovery of the new species illustrates the rich plant diversity in limestone landforms of southern Hunan once again, most of which have inconvenient transportation and dangerous terrain, and explains that we still need to conduct more in-depth research in this area.

**Figure 7.**
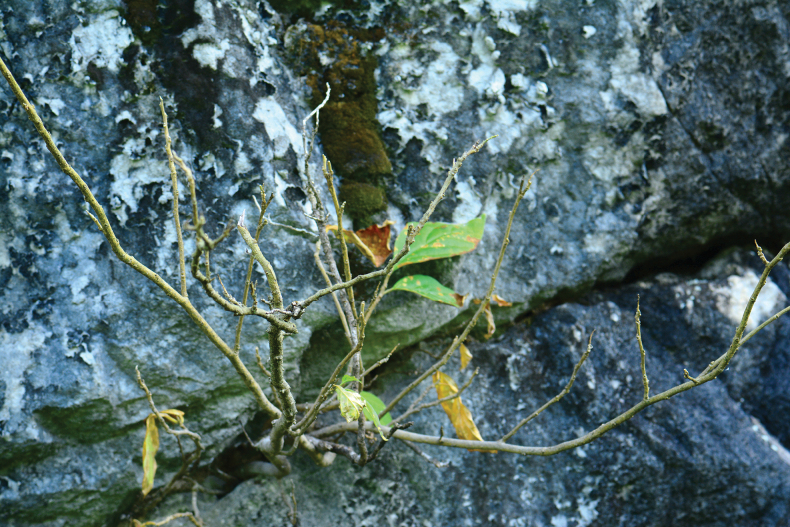
The plants of *Polygalaqii* sp. nov. had fallen leaves in July. Photographed by Jian-Jun Zhou, 21 July 2020.

This unique new species withers its leaves in July and August (Please refer to Fig. 7), which may be to adapt to the high summer temperatures in the limestone areas of southern Hunan. This phenomenon has important reference significance for studying the adaptation of plants to the environment in limestone areas. Especially in areas with well-developed karst landforms, it’s worth making a more profound study.

## Supplementary Material

XML Treatment for Polygala qii

## ﻿Additional information

### Author ORCIDs

Ang Liu https://orcid.org/0000-0001-6281-7145

Jian-jun Zhou https://orcid.org/0009-0009-9527-7152

Lei Wu https://orcid.org/0000-0003-1451-7855

### Data availability

All of the data that support the findings of this study are available in the main text.
